# Correction of post-surgical synmastia after stepwise treatment of pectus excavatum and breast asymmetry—A case report

**DOI:** 10.1016/j.jpra.2025.08.034

**Published:** 2025-09-03

**Authors:** I. Zucal, D. Schmauss, C. Parodi, Y. Harder

**Affiliations:** a Department of Plastic, Reconstructive and Aesthetic Surgery and Hand Surgery, Centre Hospitalier Universitaire Vaudois (CHUV), Rue du Bugnon 46, 1011 Lausanne, Switzerland; b Department of Plastic, Reconstructive and Aesthetic Surgery, Ospedale Regionale di Lugano, Ente Ospedaliero Cantonale (EOC), Via Tesserete 46, 6900 Lugano, Switzerland; c Faculty of Biomedical Sciences, Università della Svizzera Italiana (USI), Via Giuseppe Buffi 13, 6900 Lugano, Switzerland; d Faculty of Biology and Medicine, University of Lausanne (UNIL), Rue du Bugnon 21, 1011 Lausanne, Switzerland

**Keywords:** Breast implants, Custom made implant, Funnel chest, Mammoplasty, Pectus excavatum, Surgical mesh

## Abstract

We present a case of a 30-year-old female patient requiring correction of post-surgical synmastia after stepwise treatment of pectus excavatum (funnel chest) and breast asymmetry (right < left).

The funnel chest was first corrected with a 3D custom-made implant, placing the cranial part of the implant in a sub-muscular pocket. Asymmetric breast hypotrophy was corrected by means of subfascial breast augmentation one year later.

During follow-up, synmastia developed due to the lack of soft tissue interface between the distal part of the custom-made thoracic implant and the breast implants. Corrective surgery exposed the subjacent sternal periosteum excising a central wedge of the custom-made implant, allowing for quilting sutures between breast skin and periosteum to reconstruct the intermammary fold. Breast asymmetry was corrected using different implants that were hold in place by a synthetic mesh, acting as an inner bra.

At the 1-year follow-up, breast symmetry was observed with a preserved intermammary fold.

## Introduction

Pectus excavatum or funnel chest deformity is one of the most common thoracic deformities.^1^ Depending on its severity, either invasive chest wall surgery, such as Nuss or Ravitch procedure^2^ or custom-made pre-pectoral silicone implants are offered.^3^ Scoliosis may be associated with funnel chest deformity, often resulting in an aggravation of the chest wall deformity, sometimes even requiring orthopedic correction. If additional breast asymmetry is present, the visible volume difference of the two breasts may be even worse and associated with converging nipples. This combination of malformations is particularly challenging for the surgeon and may require a stepwise approach, since correction of one malformation can significantly impact the outcome of the correction of the other pathology.

This case report aims at describing the stepwise correction of a funnel chest deformity followed by breast asymmetry, resulting in medial breast implant migration or synmastia.

### Patient and methods

A 30 years-old, female patient presented with a combined malformation consisting of an asymmetric pectus excavatum (funnel chest deformity) that was more prominent on the right side (Cartoski IIb), scoliosis and breast hypotrophy, associated with volume asymmetry (right < left) (Figure 1). Scoliosis did not require orthopedic correction, but correction of both the chest wall and breast deformity was requested. The patient gave written consent to use her clinical and photographic data for publication and the case report was conducted according to the STROBE guidelines.

**Figure 1:**
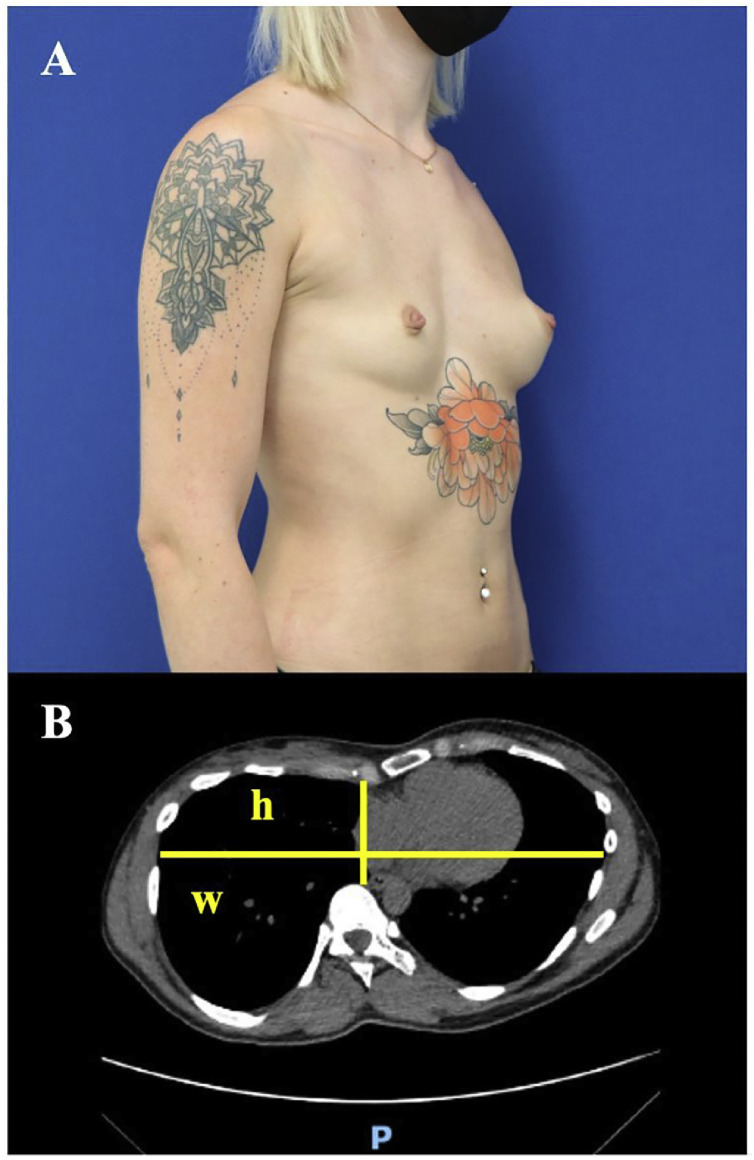
Pre-operative state. A: ¾ view of the funnel chest deformity that is more pronounced on the right side, enhancing the pre-existing volume asymmetry of the breast (right > left). B: CT scan section that shows the concavity at the caudal end of the sternum, resulting in an asymmetric deformity. Haller Index: w : *h* = 8.5 : 1.9 = 4.5.

A two-step-procedure was chosen to treat the patient:

Step 1, reconstruction of the chest wall:

The medial part of the preexisting and well-defined inframammary folds was incised in a “clam-shell”-like fashion. After identification of the lower and medial part of the pectoralis major muscles, the medial insertions were detached from the sternum to dissect the implant pocket. Following hemostasis and irrigation, suction drains and the custom-made implant (Sebbin, France) were introduced into the sub-muscular pocket and the pectoralis major muscles were joined medially with absorbable sutures. Additionally, quilting sutures were used at the median sternal line between the pectoralis major muscles and the subdermal layer of skin in the pre-sternal area cranially. Layered sutures of the skin incisions were performed.

Step 2, correction of the breast asymmetry:

Incision was made extending the pre-existing scar laterally. An epi‑pectoral, subfascial breast pocket was created bilaterally. Sizers were used to define the volume needed for each breast. After hemostasis, irrigation and suction drain placement, the final implants were inserted (right breast: Motiva Ergonomix ERSD-320Q; left breast: Motiva Ergonomix ERSM-275Q; Establishment Labs, Costa Rica). The inframammary fold was re-fixed using absorbable sutures and layered closure of the surgical incision was performed.

Due to post-surgical synmastia, a third step became necessary:

Step 3, correction of the synmastia:

The inframammary fold-scar was excised and the previous breast implants removed. After incising the capsule underneath the breast implants, the pre-sternal chest wall implant was incised longitudinally at its caudal midline to excise a converging wedge until the pre-sternal periosteum was exposed. This allowed to further pull down the pectoralis muscles to cover the cranial part of the pre-sternal implant and redefine the medial contour of the breast or inter-mammary fold by using quilting sutures (between pre-sternal dermis and pectoralis muscles cranially and pre-sternal dermis and pre-sternal periosteum caudally). Thereafter, a bra-shaped, non-absorbable synthetic mesh (TiLOOP® Bra Pocket, PFM Medical, Köln, Germany) was used to define the new prepectoral breast implant pocket using absorbable sutures. After sizer evaluation, the final breast implants were inserted (right breast: Motiva Ergonomix ERSD-380Q; left breast: Motiva Ergonomix ERSM-300Q) into the predefined pocket supported by the mesh. Layered sutures of the surgical incisions were performed and a compressive bra as well as compressive bolstering at the intermammary fold was used for 6 weeks after surgery.

The surgical procedures are illustrated schematically in Supplementary material 1.

## Results

The patient presented with a funnel chest deformity that was dominant on the right side, aggravated by a right-convex scoliosis of the spine. Moreover, breast volume asymmetry (right < left) was enhanced by the afore-mentioned malformations. The pre-operative photo and the CT-scan of the thorax are shown in Figure 1 and Supplementary material 2. As the first surgical step, a custom-made pre-sternal implant was used.

After the first surgery, the funnel chest deformity was successfully reconstructed (Supplementary material 3), creating an adequate and symmetric base for subsequent combined symmetrization and augmentation of the breasts. This motivated the patient to undergo the second surgery 4 months later. Though, after surgical correction of breast asymmetry, the patient developed medial displacement of both implants, resulting in synmastia (Figure 2 and Supplementary material 4).

**Figure 2:**
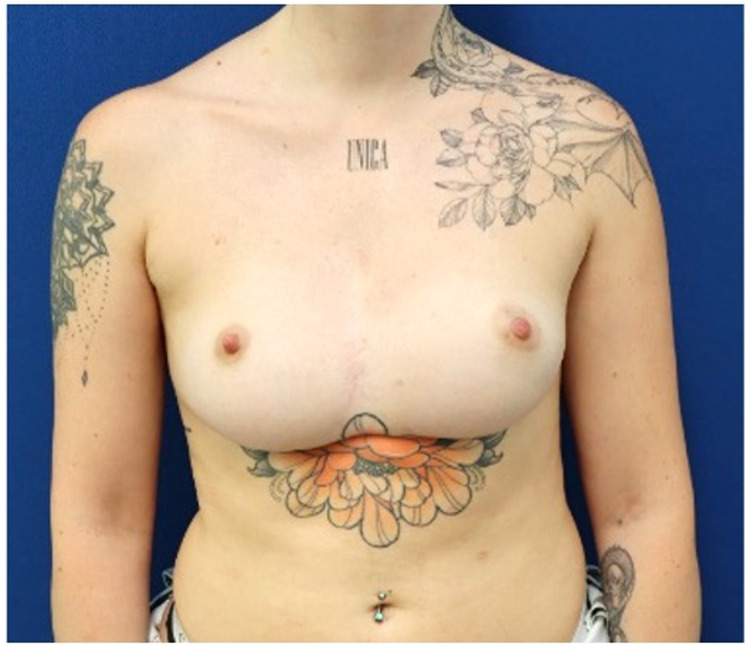
Postoperative state after correction of breast asymmetry (Second stage surgery). Postsurgical synmastia is observed at 6 weeks after surgery.

Surgical revision was performed another 10 months later: to prevent a recurrent displacement of the breast implants, it was decided to use a mesh acting as an “inner bra or hammock.” Moreover, the custom-made pre-sternal implant was split longitudinally in its distal and median part, exposing the sternal periosteum. This allowed to redefine the intermammary fold using quilting sutures, without any signs of displacement at the 12 months follow-up. Furthermore, the placement of the breast implant on the right onto the CMI-implant, i.e. the corrected concavity due to the right-dominant funnel chest deformity, resulted in a lateralization of the right nipple changing from a convergent to a divergent one (Figure 3 and Supplementary material 5 & 6). To correct volume asymmetry of the breasts, round implants of similar width, yet different projection were used.

**Figure 3:**
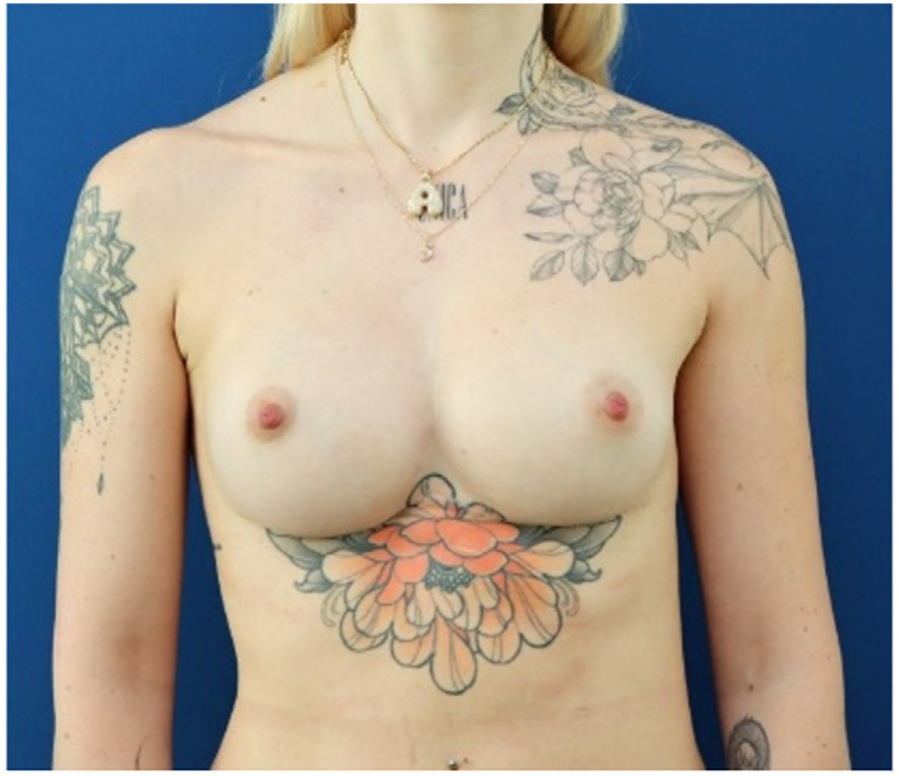
Postoperative state after correction of synmastia (Third stage surgery). After mesh insertion, breast implant exchange, and intermammary fold reconstruction, a symmetric and stable result is achieved, as seen at the 1-year follow-up.

## Discussion

Considering the rather infrequent prevalence of funnel chest deformity and its potential combination with severe scoliosis and/or breast asymmetry, there is no standardized surgical approach defined so far. After balancing pros and cons, we opted for a stepwise approach. The reconstitution of the right dominant funnel chest deformity aimed at restoring a symmetric platform to enable a “conventional” breast augmentation. This allowed to correct—though in a second stage— volume asymmetry of the breast. However, two factors resulted in medial migration of the breast implants and eventually postsurgical synmastia:

1. Insufficient adherence of the caudal intermammary skin that could not re-adhere due to the absence of pectoralis muscles, but the presence of a thin capsule covering the underlying chest wall implant and
2. Almost inexistent thin capsule around the breast implants, not capable of maintaining them in place. To avoid recurrence of progressive medial implant-displacement, we decided to add pre-shaped, non-absorbable meshes that could maintain the implants in place, once correctly placed within the surgically prepared pocket. Alternatively, one could think of using macrotextured breast implants that adhere to the surrounding tissues. The latter are either not used anymore due to the fact of potential development breast implant associated anaplastic large cell lymphoma^4^ or coated with polyurethane (PU). PU-coated implants however need to be perfectly placed from the very beginning until they have had time to “in-grow” with the surrounding tissues.^5^

In the reported case, the insertion of a surgical mesh was chosen to prevent synmastia recurrence, as it anticipates implant migration.^6^ So far, this mesh has predominantly been used in immediate, prepectoral breast reconstruction after mastectomy, both if there is a mismatch between the pocket’s width and the implant’s base and to relieve the distal part of the mastectomy flap.^6^, ^7^, ^8^ Regarding these meshes, an acceptable complication rate is reported in literature,^7^^,^^9^ with seroma being the most common complication.^7^^,^^9^ Moreover, BREAST-Q analyses showed a high patient satisfaction in the follow-up^8^ which is in line with this report that confirms a stable breast implant situation and highly satisfied patient at 1 year.

In conclusion, combined chest wall and breast deformity are challenging for the surgeon and therefore a stepwise procedure may be the better solution compared to all-in-one surgery. Furthermore, restoring an adequate and symmetric platform for subsequent breast implant placement, is necessary to correct non-physiological nipple convergence often associated with funnel chest deformity. Nevertheless, even after a staged procedure, implant-related complications may occur and therefore require “thinking out of the box” depending on the complication. In this regard, the use of surgical meshes may be a solution, since the breast implants that have been used in this case neither adhere to the surrounding tissues, nor develop a stable capsule.

## Declaration of competing interest

None declared

## Appendix Supplementary materials

Image, application 1
