# Supplementary material for: Correction of post-surgical synmastia after stepwise treatment of pectus excavatum and breast asymmetry—A case report
Source: JPRAS Open. 2025 Sep 3;46:494–9. doi: 10.1016/j.jpra.2025.08.034 (PMC12616097; doi:10.1016/j.jpra.2025.08.034)
Supplement: Supplementary file 1 [file mmc1.pdf]

## Supplementary content

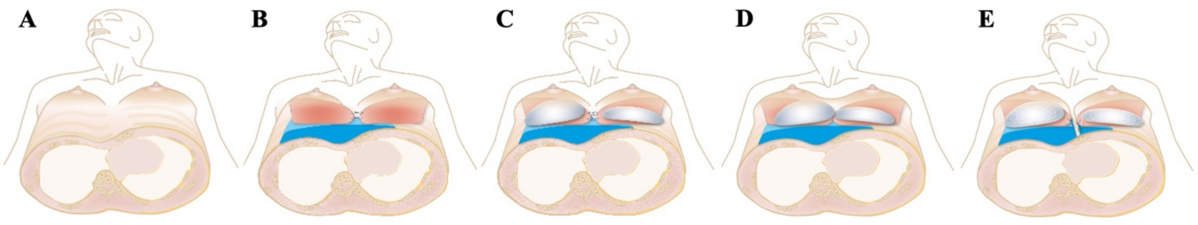


**Supplementary material 1. Schematic reproduction of the stepwise surgical procedure.** A: Pre-operative status with right-sided, asymmetric funnel chest indicating convergent nipples and breast volume deformity (right < left). B: Post-operative status after 1st surgery: insertion of the custom-made implant in a subpectoral plane, partially detaching the medial muscular insertions to create the pocket cranially before re-adapting the two muscles. C: Post-operative status 4 weeks after 2nd surgery, i.e. subfascial, but prepectoral breast augmentation. D: Bilateral medial migration of the breast implants due to the lack of adherence between breast implants and surrounding tissues ("tissue interface") creating postsurgical synmastia. E: Post-operative status after surgical correction, including excision of a central wedge of the custom-made implant, medial sutures to withstand cranio-lateral retraction of the pectoralis muscles, repositioning of the breast implants within an inner bra-like non-resorbable synthetic mesh and quilting sutures between the exposed sternal periosteum and subdermal layer of the medial breast skin to redefine the cleavage area. Note the newly built "platform" for symmetric breast implant placement that allowed spontaneous lateral nipple displacement on the right (A-E).

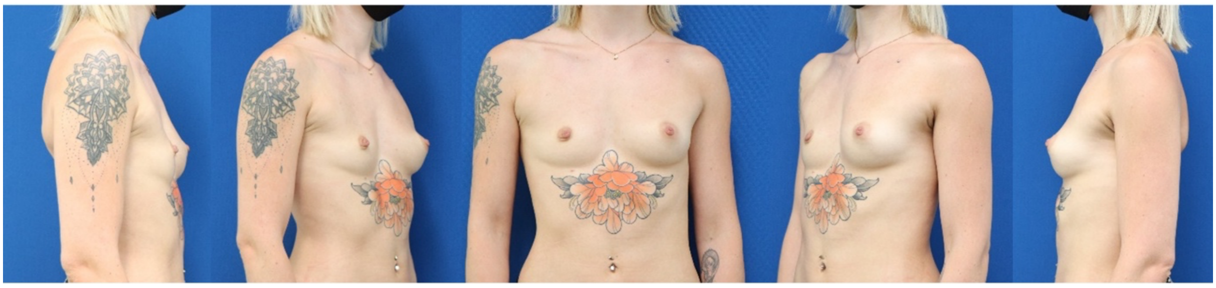

**Supplementary material 2. Pre-operative state.** Volume asymmetry of the breast (right < left) well visible in the right profile view and funnel chest deformity. Note the non-diverging nipples in the front view, indicating the presence of an asymmetric funnel chest deformity that is right-dominant.

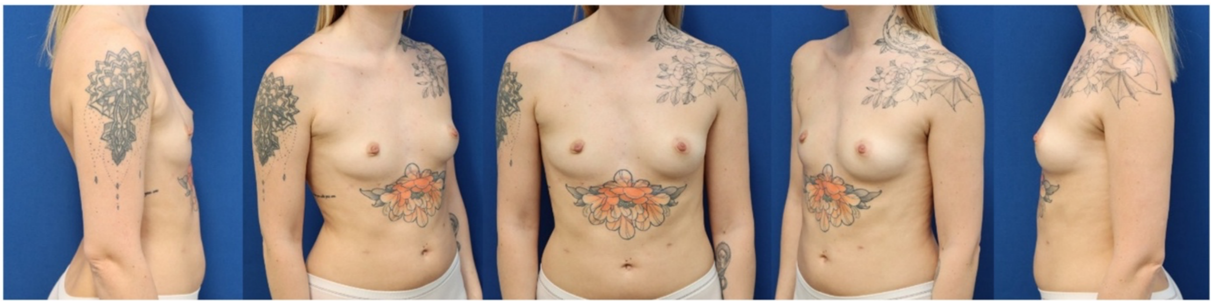

**Supplementary material 3. Post-surgical result after 1st-stage-surgery.** Correction of the funnel chest deformity that is best seen by the slight lateral migration of the right nipple, indicating that the right-

dominant funnel chest deformity has been corrected, eventually creating a symmetric and uniform "platform" for subsequent breast implant positioning.

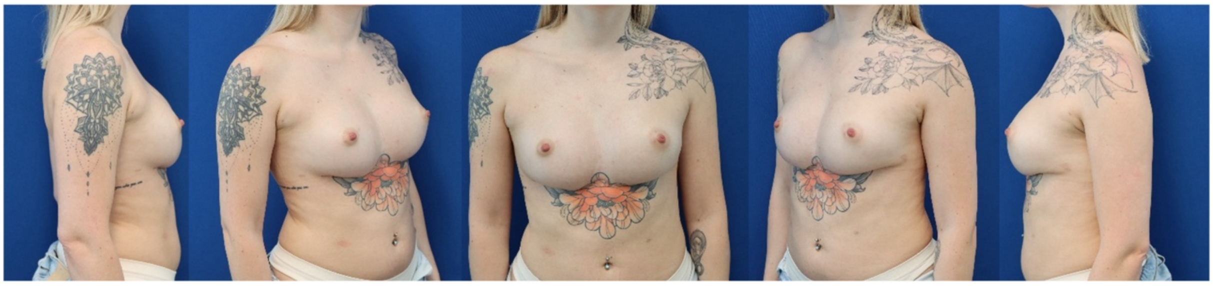

**Supplementary material 4. Post-operative result after 2nd-stage-surgery.** Medial migration of both breast implants creating post-surgical synmastia with complete disappearance of the inter-mammary fold.

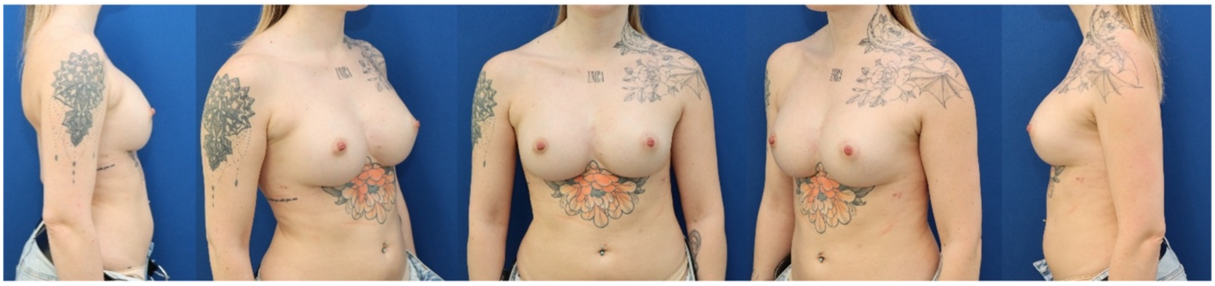

**Supplementary material 5. Post-operative result after corrective, 3rd-stage-surgery.** At 1 year of follow-up, stable implant position and well defined inter- and infra-mammary fold, as well as breast symmetry is observed. Note the lateralization of the nipples with decreased bilateral horizontal distances between lateral aspect of the thorax and the nipples.

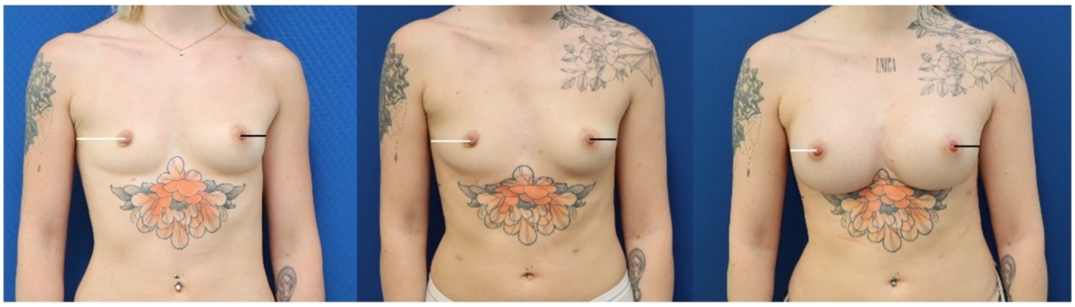

**Supplementary material 6. Pre- and post-operative result.** Note the asymmetric nipple position before funnel chest correction, showing the right nipple medially displaced due to the right-dominant chest wall concavity. Step-wise lateral displacement of the right nipple following the insertion the customized implant to correct first chest wall asymmetry and eventually breast implants to correct hypotrophy and asymmetry. Note the gradual displacement of the right nipple with a significantly decrease of the horizontal distance between the lateral aspect of the thorax and the nipple (indicated by the white line), whereas on the left, this distance remained the same (black line). This indicates that the customized implant could restore symmetry of the bony thorax and therefore result in a symmetric "platform" for the subsequent breast implants, resulting in a converging nipple to become a diverging nipple.
